# Biochar‐Compost From Cashew Apple Residue as a Soil Amendment for Cashew Cultivation in Ghana

**DOI:** 10.1002/pei3.70138

**Published:** 2026-03-10

**Authors:** Elvis Frimpong Manso, Michael Teye Barnor, Mutala Edem Baba, Wisdom Edem Anyomi

**Affiliations:** ^1^ Cocoa Research Institute of Ghana, Bole Substation Bole Ghana; ^2^ Council for Scientific and Industrial Research‐Soil Research Institute Kwadaso Ghana

**Keywords:** biochar, biochar‐compost, cashew apple residue, Guinea savannah, soil amendment

## Abstract

The study investigated the effect of different compost types on the growth performance of cashew ( *Anacardium occidentale* L.) varieties cultivated in a Lixisol from Northern Ghana. Six compost formulations were prepared using various combinations of poultry manure, cow dung, biochar, and cashew apple residue at different mixing ratios. The experimental design was a completely randomized block design arranged in a 2 × 6 factorial structure with three replicates. Three cashew varieties were planted at a spacing of 10 m × 10 m and amended with compost types at a rate of 50 kg ha^−1^. Growth parameters were recorded over two seasons, while nut yield was recorded in year 2. Results showed that cashew apple residue combined with biochar and poultry manure produced composts with higher organic carbon (OC) and total nitrogen (N) than the controls. In the first year, variety B3T101 treated with either cow dung or a mixture of poultry manure, biochar, and cashew apple residue in a 2:1:1 ratio recorded significantly (*p* < 0.05) higher heights (205.70 and 193.00 cm, respectively). However, in year 2, all compost types had statistically similar effects on plant height (*p* < 0.05). Variety B3T57 treated with poultry manure alone and biochar‐poultry manure‐cashew apple residue had statistically similar (*p* < 0.05) nut yield of 803.70 and 872.30 g, respectively. The findings demonstrate that cashew apple residue is as valuable as the traditional decomposed cow dung and poultry manure used in cashew farming for use as an organic fertilizer in cashew farming.

## 1 Introduction

Cashew ( *Anacardium occidentale* L.) production is vital to Ghana's economy (SRID 2022). The cultivation occurs in three agro‐ecological zones (the Transitional, the Northern, and the Coastal Savannah) (Frimpong 2016), characterized by nutrient‐poor soils with low moisture retention (Nartey et al. 1997; Fianko et al. 2023). Although cashew can thrive in various soil types, its growth is hindered by poor soil conditions, necessitating soil fertility amendments for better yields (Rupa et al. 2013). The progressive decline of soil fertility in cashew farms is owing to the inability of peasant farmers to procure over‐reliant yet expensive and scarce inorganic fertilizers. Furthermore, there is a lack of information on recommended fertilizer application rates and their consequential effects on cashew plantation establishment (Akanbi et al. 2013). As a result, most cashew plantations have low nut yields (Dadzie et al. 2014; Frimpong 2016), realizing only about 15.67% of the potential nut yield (SRID 2022).

The application of organic fertilizers, such as compost, has been identified as one of the strategies to improve soil quality in Northern Ghana (Sulemana et al. 2021; Fianko et al. 2023). Organic materials can alleviate the low nutrient levels in these soils by releasing plant essential elements and improving other soil properties (Tandy et al. 2009; Akumah et al. 2021). Compost has been reported to increase plant biological activity and soil nutrient availability, thereby enhancing crop productivity (Tandy et al. 2009; Suvendran et al. 2025). In cashew, Ipimorotimi and Akanbi (2015) reported that organic fertilizer treatments produced better growth responses than inorganic fertilizers. Consequently, there has been increased advocacy for the use of organic fertilizers to enhance the carbon and nutrient content of these fragile soils (Sulemana et al. 2021; Fianko et al. 2023).

Most farm residues in the Guinea savannah zone of Ghana are either burnt or removed to feed livestock at the end of the farming season (Fianko et al. 2023), making the availability of compostable feedstock a challenge. The expansion of agricultural production on these soils (SRID 2022) implies that a larger volume of organic fertilizers is required to enrich the soils for optimum yield. This may create competition for the few feedstocks, such as maize stover, groundnut husk, kitchen waste, cow dung, and poultry manure that are available and commonly used among farmers, causing a foreseen price surge and shortage.

Similarly, cashew apple residue provides an underutilized resource with significant potential for composting, given the large volumes produced (Akyereko et al. 2023). Ghana's cashew industry has expanded from 87,000 ha in 2012 to 214,000 ha in 2021 (SRID 2022) and annually produces about 1,530,000 MT of cashew apples (Akyereko et al. 2023), which currently hold low economic value and mostly go to waste without alternative uses. Cashew apple residue, like other organic materials, contains plant nutrients (Batham et al. 2013) that, once composted, could be made available to cashew plants, especially in the Guinea savannah zone, where soil organic carbon and nutrient levels are very low. Indeed, Ganesan et al. (2020) reported increased cashew growth after amendment with vermicompost made from cashew apple waste. A major challenge of cashew apple residues is their high organic acid content (pH ranged from 3.5 to 4.8) (Marc et al. 2012; Salehi et al. 2020), which may further acidify the compost or soil environment. Since most soils in the Guinea savannah are already acidic, any amendment should raise soil pH and improve nutrient availability. In this respect, biochar has been shown to increase the pH of amended media (Novak et al. 2009; Frimpong Manso et al. 2019). Converting cashew apple residues into biochar compost for farm application can help address nutrient depletion and close the nutrient gap (Fianko et al. 2023) in cashew plantations. Biochar can adsorb nutrients and dissolved organic matter during composting, enhancing the quality of the final product (Prost et al. 2013). The composted biochar can be used as a soil amendment, offering benefits such as improved nutrient use efficiency, higher crop yields, and long‐term soil carbon sequestration (Schulz et al. 2013). For example, Agegnehu et al. (2015) observed increased peanut yield, soil water content, and soil nutrients on Ferralsols following application of composted biochar. Additionally, it has been reported that incorporating biochar during composting can potentially boost the phosphorus content of the compost (Sulemana et al. 2021; Fianko et al. 2023), enhance microbial growth (Jindo et al. 2012), improve aeration (Zhang et al. 2014), and reduce leaching during composting (Steiner et al. 2010; Akumah et al. 2021). An increase in nutrient retention within compost has been linked to the enhanced surface oxidation of added biochar (Steiner et al. 2010).

Variety plays a crucial role in achieving high cashew yields due to differing requirements and responses to fertilizer application (Arora et al. 2001; Nipa et al. 2013). Cashew varieties can differ in nutrient use efficiency (Rodgers and Barneix 1988) and internal nutrient utilization (Van Sanford and MacKown 1986), yet new improved varieties are released without tailored fertility recommendations (Wissum et al. 2009). Therefore, it is wise to evaluate how different cashew varieties respond to soil fertility amendments.

With all the possible uses of cashew apple residues (Patade et al. 2020) and the high volumes of cashew apple residues (229,500 MT) that are generated annually, the material after harvesting of the nuts is left on the farmers' field without any proper disposal, creating an environmental nuisance and posing a potential threat to plants and human health; however, these residues can be channeled into composting with biochar as a means to return nutrients into the soil and enhance the growth of cashew plantations. Research focusing on the use of biochar composting to specifically enhance the pH and harness nutrients from cashew apple residues and cashew varietal response to soil fertility amendments in Ghana is lacking. This study investigated the potential of using cashew apple residue to produce biochar‐compost for cashew farming in Ghana.

## 2 Materials and Methods

### 2.1 Characteristics of Experimental Location

The study was undertaken at the Bole substation of the Cocoa Research Institute of Ghana (231° SW at 090°0′43′ N; Long 20°32′11″ W; Alt 320 m) from 2022 to 2025. The area is under Ghana's Guinea savannah agro‐ecological zone with a unimodal rainfall pattern, usually from May to October. Annual rainfall in this zone ranges between 800 mm and 1200 mm, with maximum temperatures up to 42°C during the dry season (GMET 2020). The soils are classified as Lixisols (Dedzoe et al. 2001). These soils are reddish‐brown in color, moderately well‐drained with moderately deep (100–150 cm) topsoil with concretions. These soils have low cation exchange capacity and a high base saturation. These soils are fairly susceptible to erosion and compaction due to their poorly developed structures. They have vegetation cover mainly of grasses and shrubs with dispersed trees (Dedzoe et al. 2001).

### 2.2 Routine Soil Analyses

Soil and compost pH were determined electrometrically in water (1:2.5 and 1:10, respectively). The organic carbon content of soil and compost types was determined using the Walkley and Black (1934) wet oxidation method. The total nitrogen content of the soils and the compost was determined using the Kjeldahl (1883) method. Available phosphorus was determined using the Mehlich method (1984). Exchangeable bases were determined by the ammonium acetate method (Black 1965), and the filtrates were analyzed on an atomic absorption spectrometer version iCE 3300 (Thermo Scientific, Waltham, Massachusetts, USA). Exchangeable acidity was determined using the KCl extraction method. Soil particle size was determined using the Bouyoucos hydrometer method.

### 2.3 Experimental Design and Treatments

Rice husk biochar (RHB) was produced using a Kuntan kiln at an average pyrolysis temperature of approximately 425°C. The biochar was subsequently used in the composting of Cashew Apple Residue (CAR), Cow Dung (CD), and Poultry Manure (PM) at 4 mixing ratios on a v/v basis.

Four compost mixtures were formulated as follows:

- Compost 1: PM + RHB (2:1)
- Compost 2: PM + RHB + CD (2:1:1)
- Compost 3: PM + RHB + CAR (2:1:1)
- Compost 4: PM + RHB + CAR (2:2:1)

In addition to the mixed heaps, two single‐source compost treatments were prepared:

- Compost 5: CD only
- Compost 6: PM only

All compost heaps were constructed to uniform dimensions of 2 m × 2 m × 3 m and were composted to maturity. The cashew apple residue was collected from the cashew plantation farm of the Cocoa Research Institute of Ghana in Bole. Cow dung and poultry manure were collected from livestock farms in surrounding communities, while rice husk was collected from a rice mill at Tamale. A field trial using a 6 × 3 factorial experiment consisting of 6 compost amendments and 3 cashew varieties was set up in a randomized complete block design (RCBD) with three replications. At the onset of the rainy season, 8‐week‐old seedlings of three promising cashew varieties were transplanted in the field at a spacing of 10 m × 10 m. Each plot received matured biochar‐compost at an application rate of 50 kg ha^−1^ (commonly practiced by the local farmers). Sole cow dung compost treatment was selected as the control treatment, owing to the common practice of using cow dung to raise cashew seedlings by the majority of farmers in the area.

### 2.4 Data Collection and Analyses

Data on stem diameter and plant height from the treatments were collected. The initial growth measurement was done just before transplanting; the second growth measurement was done 1 year after field establishment, and subsequent measurements were done at yearly intervals. Each replicate had three plants that were earmarked for growth data collection. The stem diameter of the three cashew plants was measured with digital calipers at 15 cm from the soil surface at the base of each plant. The height of these three plants was also measured from the base of each plant to the tip of the leading apex leaf using a tape measure. Nuts from the trees were collected, and the yield was estimated. The three plants from each of the three plots (a total of 9) were summed up, and an average value was determined as representative of each treatment. Initial height and diameter recorded at the time of transplanting were deducted from the measurements in years 1 and 2 to obtain the actual plant growth. Nut yield data were collected from February to March 2025. The data collected were analyzed following the procedures for analysis of Variance and treatment and interaction effects means separated using the LSD at *p <* 0.05.

## 3 Results

### 3.1 Chemical Properties of the Compost Types Used in the Experiment

The use of non‐composted cow dung in raising cashew seedlings is a common practice of cashew plantation farmers in the Guinea savannah, largely due to the inherently low fertility of soils in the region. However, composting improved the quality of the final product, both in terms of nutrient content and pH. Composting poultry manure alone (compost 6) proved superior in nutrient concentrations to cow dung compost alone (compost 5), except for carbon content, with both composts having an alkaline pH (Table 1). This highlights the prospects for poultry manure as an alternate organic amendment when available in commercial quantities. Co‐composting the poultry manure with rice husk biochar (compost 1) increased the pH of the final product with a consequential increase in C and Mg contents (Table 1). However, this had a negligible effect on N content and possible dilution of the P, K, and Ca contents. The addition of cow dung to the composting mix (compost 2) resulted in increased C, while slightly reducing concentrations of P, Ca, and Mg, whereas pH, N, and K were little affected. Replacing cow dung with cashew apple residue in the composting mix (compost 3) decreased the pH, C, and K contents of compost. Whereas N content was similar, the concentrations of P, Ca, and Mg increased by 1.32, 1.60, and 1.39‐fold, respectively. An increase in the proportion of rice husk biochar (compost 4) resulted in similar pH and P with increased C and N contents. The concentrations of basic cations decreased in compost 4 (Table 1).

**TABLE 1:** Chemical properties of the compost types used in the experiment.

Compost	pH	C	N	P	K	Ca	Mg
		%					
1	8.12 ± (0.96)	23.00 ± (1.23)	1.74 ± (0.20)	1.26 ± (0.06)	1.50 ± (0.01)	5.37 ± (0.72)	1.90 ± (0.18)
2	8.09 ± (1.23)	24.50 ± (2.15)	1.82 ± (0.13)	0.91 ± (0.01)	1.63 ± (0.12)	4.40 ± (0.88)	1.12 ± (0.06)
3	8.31 ± (2.01)	22.50 ± (1.19)	1.85 ± (0.03)	1.20 ± (0.05)	1.39 ± (0.03)	7.05 ± (0.41)	1.56 ± (0.08)
4	8.18 ± (0.15)	25.50 ± (2.05)	2.66 ± (0.09)	1.24 ± (0.13)	1.20 ± (0.04)	5.45 ± (0.88)	1.22 ± (0.06)
5	7.31 ± (1.23)	23.50 ± (1.12)	1.71 ± (0.01)	0.23 ± (0.01)	1.64 ± (0.11)	1.36 ± (0.22)	0.97 ± (0.02)
6	8.05 ± (0.98)	20.00 ± (1.85)	1.76 ± (0.03)	1.43 ± (0.03)	2.00 ± (0.08)	8.50 ± (0.16)	1.65 ± (0.08)

*Note:* Figures in brackets are standard errors.

Abbreviations: C = carbon, Ca = calcium, K = potassium, Mg = magnesium, N = nitrogen, P = phosphorus.

### 3.2 Characteristics of the Soil Used in the Experiment

Some physicochemical properties of the soil used are shown in Table 2. The soil had a moderately acidic pH of 5.6, which is higher than the critical minimum required for cashew production (Duncan 2001). The soil had a high sand content (85.2%) compared to the clay and silt content. The soil organic carbon content was 1.01%, exceeding the critical minimum threshold for cashew cultivation (Duncan 2001). The cation exchange capacity (3.71 cmol kg^−1^) and available P (3.34 mg kg^−1^) of the soil were below the critical minimum values of 10 cmol kg^−1^ and 13 mg kg^−1^, respectively (Duncan 2001), and therefore agricultural practices such as compost application are required to increase the soil CEC and available P to ensure optimum and sustainable cashew production. The soil had low K, Mg, and Na content, with relatively higher Ca content (Table 2), all of which were above the critical minimum levels required for cashew production (Duncan 2001). Additionally, the soil had a low exchangeable acidity of less than 15%, making it suitable for cashew production, provided other conditions are ideal.

**TABLE 2:** Some physicochemical properties of the soil used in the experiment.

Soil parameters	
Sand (%)	85.20
Silt (%)	4.14
Clay (%)	10.66
Textural class	Loamy sand
pH	5.64
O.C (%)	1.01
T.N (%)	0.09
Avail. P (mg kg^−1^)	3.34
Ca (cmol kg^−1^)	2.29
Mg (cmol kg^−1^)	0.71
K (cmol kg^−1^)	0.28
Na (cmol kg^−1^)	0.01
T.E.B (cmol kg^−1^)	3.29
Exch. Acidity (cmol kg^−1^)	0.42
CEC (cmol kg^−1^)	3.71
% Acidity	11.32

Abbreviations: Avail. P = available phosphorus, Ca = calcium, CEC = cation exchange capacity, Exch. = exchangeable, K = potassium, Mg = magnesium, Na = sodium, O.C = organic carbon, T.N = total nitrogen, T.E.B = total exchangeable bases.

### 3.3 Effect of the Compost Application on Cashew Plant Height

The effect of compost application on the height of the different cashew varieties over two growing seasons is shown in Table 3. In the first growing season, the cashew variety B3T101 treated with compost 5 had the highest plant height, which was insignificantly (*p* < 0.05) different from varieties B3T101 treated with compost 3 (193.00 cm), B3T101 and B3T80 treated with compost 2, B3T80 treated with compost 3, B3T80 amended with compost 4, B3T80 amended with compost 5 and B3T101, B3T80 amended with compost 6 (Table 3). On the other hand, variety B3T57 (132.30 cm) treated with compost 3 recorded the significantly (*p* < 0.05) lowest plant height. However, it was statistically (*p* < 0.05) similar to all the varieties treated with compost 1, B3T101 and B3T57 treated with compost 4, and B3T57 amended with both composts 5 and 6.

**TABLE 3:** Effect of compost application on the height of the cashew varieties.

Treatment	Compost type	Variety	Height (cm)	
			Year 1	Year 2
1	Compost 1	B3T101	152.70a	215.10a
2		B3T57	139.00ab	188.70a
3		B3T80	159.00ab	213.60a
4	Compost 2	B3T101	168.70bc	236.60a
5		B3T57	165.10b	207.00a
6		B3T80	192.50bc	227.80a
7	Compost 3	B3T101	193.00c	219.00a
8		B3T57	132.30a	191.90a
9		B3T80	179.00bc	233.20a
10	Compost 4	B3T101	152.10a	212.40a
11		B3T57	142.60ab	196.30a
12		B3T80	176.10bc	222.20a
13	Compost 5	B3T101	205.70c	237.40a
14		B3T57	156.00ab	208.80a
15		B3T80	168.10bc	215.20a
16	Compost 6	B3T101	166.70bc	229.40a
17		B3T57	159.10ab	222.70a
18		B3T80	178.40bc	230.20a

*Note:* Means with the same alphabet are statistically similar (*p* < 0.05).

**FIGURE 1:**
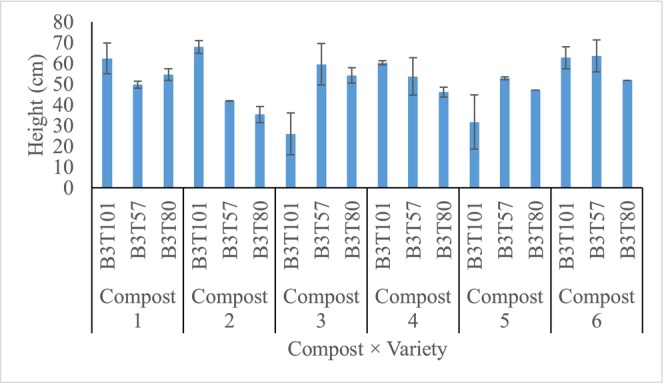
Graph of height increment from year 1 to year 2. Bars = standard error of means.

Although there were insignificant (*p* < 0.05) differences in the plant height in the second year (Table 3), the different treatments recorded significant (*p* < 0.05) differences in the height increment from year 1 to year 2 (Figure 1). Cashew varieties B3T80, B3T101, and B3T57 treated with composts 2, 3, and 5, respectively, had the least similar (*p* < 0.05) height increment during the 2 years of the study. Whilst variety B3T57 treated with compost 2 recorded the highest height increment from the first to the second year (Figure 1).

### 3.4 Effect of the Compost Application on Plant Diameter

**FIGURE 2:**
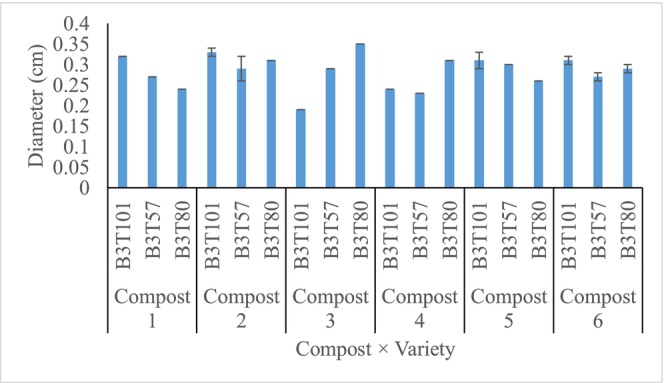
Graph of diameter increment from year 1 to year 2. Bars = standard error of means.

The effect of the compost application on the stem diameter of the three cashew varieties over two growing seasons is shown in Table 4. All treatments recorded similar plant diameters (*p* < 0.05) in both years of plant growth. However, the different treatments showed differences in diameter increments, with variety B3T80 treated with compost 1 showing the largest increment. This was similar to variety B3T101 treated with compost 1, B3T101 and B3T80 treated with compost 2, B3T80 treated with compost 4, B3T101 and B3T57 treated with compost 5, and B3T101 treated with compost 6 (Figure 2). Variety B3T101 treated with compost 3 had the smallest stem diameter increment.

**TABLE 4:** Effect of compost application on the diameter of cashew varieties.

Treatment	Compost type	Variety	Diameter (cm)	
			Year 1	Year 2
1	Compost 1	B3T101	0.50a	0.82a
2		B3T57	0.39a	0.66a
3		B3T80	0.49a	0.73a
4	Compost 2	B3T101	0.48a	0.81a
5		B3T57	0.41a	0.70a
6		B3T80	0.46a	0.77a
7	Compost 3	B3T101	0.54a	0.73a
8		B3T57	0.37a	0.66a
9		B3T80	0.49a	0.84a
10	Compost 4	B3T101	0.51a	0.75a
11		B3T57	0.40a	0.63a
12		B3T80	0.43a	0.74a
13	Compost 5	B3T101	0.56a	0.87a
14		B3T57	0.39a	0.69a
15		B3T80	0.45a	0.71a
16	Compost 6	B3T101	0.50a	0.81a
17		B3T57	0.47a	0.74a
18		B3T80	0.45a	0.74a

*Note:* Means with the same alphabet are statistically insignificant (*p* < 0.05).

Detailed raw data regarding the impact of different compost types on the growth parameters of various cashew varieties across growing seasons can be found in the Supporting Information section (Table S1).

### 3.5 Effect of Compost Treatments on the Early Nut Yield of the Cashew Varieties

**FIGURE 3:**
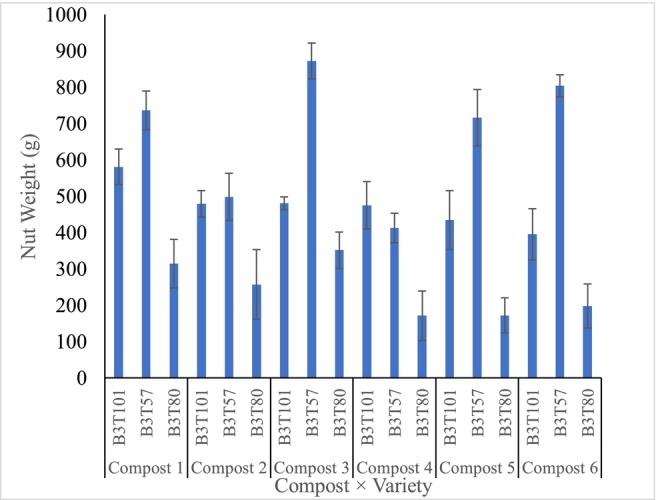
Effect of the compost amendments on the nut weight of the cashew varieties. Bars = standard error of means.

Early nut yields of the cashew varieties are presented in Figure 3 below. The B3T80 treated with compost 4 had the lowest yield; this yield was not significantly different (*p* > 0.05) from that of B3T80 treated with composts 5 and 6. The B3T57 treated with compost type 3 had the highest nut yield of 872.30 g, which was statistically similar (*p* < 0.05) to the same variety treated with composts 1, 5, and 6. However, it was significantly higher than the other treatments (*p* < 0.05). Generally, variety B3T57 recorded the highest nut weight across the different compost treatments.

## 4 Discussion

### 4.1 Chemical Properties of the Different Types of Compost Used

Carbon is an important element in ensuring soil health and sustainable food production. Compost 4 had a higher carbon content that can be attributed to the incorporation of biochar. A trend similarly noted in the other compost treatments that included biochar. This is further confirmed by the findings of Aboagye et al. (2022), who reported increased carbon content of compost with increasing biochar. The high carbon content of compost 5, despite the absence of biochar, is likely a result of the high‐fiber diet of cattle, predominantly composed of forage grasses and shrubs, which are typically high in fiber content. The alkaline pH of the compost types is typical of most mature compost (Aboagye et al. 2022) due to the release of basic cations and concomitant volatilization of ammonia during decomposition. The addition of biochar, especially to the compost heaps where cashew apple residue was incorporated, could contribute to the higher pHs recorded. These observations are in agreement with the findings of Sulemana et al. (2021), Rondon et al. (2007), Van Zwieten et al. (2007), and Fianko et al. (2023). In addition, the presence of negatively charged functional groups such as phenol, carboxyl, and hydroxyl on biochar surfaces can adsorb H^+^, thereby lowering their concentration with a consequential rise in compost pH (Chintala et al. 2014). The pH of the various compost types was alkaline, particularly those incorporated with biochar, highlighting their potential as liming agents for improving the pH of acidic soils. The high pH, coupled with the high C and nutrient concentrations, makes the various composts ideal for selection as organic amendments in tropical soils such as Lixisols. It is worth noting that as the biochar quantity increased, the N content of the resulting compost increased. This may be a result of the high nutrient retention capacity of biochar, which aided in minimizing N losses during composting. Similar trends have been reported by Chan and Xu (2009), Widowati and Asnah (2014), and Aboagye et al. (2022), that biochar addition to compost has the ability to increase the N content of compost through the retention of NH_4_ and the inhibition of ammonium transformation. The addition of biochar did not necessarily increase the available P content of the compost types, contrary to the findings of Sam (2014), Akumah et al. (2021) and Fianko et al. (2023), who reported increased available P content following the addition of biochar to composting. The feedstock types used in the production of the compost might explain the cation content of the end product. The high Ca, Mg, and K content of compost 6 and biochar‐composed composts make them suitable for application on acidic soils and the cultivation of tree crops such as cashew. These cations might also be responsible for the fixation of the large amounts of released P during composting.

### 4.2 Physico‐Chemical Properties of the Soil Used

The high sand fraction of the soil might predispose the soil to nutrient leaching and erosion. The pH of the soil falls within the moderately acidic class (USDA 1999) and is within the optimal pH range of 5.5 to 6.5 for cashew cultivation (Duncan 2001). The relatively low organic carbon content of the soil can be attributed to the sparse vegetative cover and high rates of organic matter mineralization, driven by elevated ambient temperature. Furthermore, the annual incidence of bushfires, a common phenomenon in the area, may have contributed to the depletion of soil organic matter and plant nutrients. The soil had low CEC, which is typical of most tropical soils due to the prevalence of low activity 1:1 clay minerals such as kaolinite. Additionally, the high sand content and low organic carbon content further explain the low CEC of the soil. Likewise, the low total N, together with the low basic cation content, can be associated with the low CEC and organic carbon content. Soil organic matter and CEC serve as indicators of soil fertility, and therefore, soil fertility increases with increased CEC and organic carbon content and *vice versa*. The low pH and organic carbon might be the reason for the low available P content of the soil. It has been reported that at low pH, most tropical soils tend to fix P, making the element unavailable for plant uptake (Nartey et al. 1997; Fianko et al. 2023). Generally, the soil possesses chemical properties that make it suitable for growing cashew; however, soil fertility management is required to enhance fertility and also increase production of the tree crop.

### 4.3 Effect of Compost Application on Cashew Growth and Nut Yield

The application of various compost types significantly enhanced cashew plant growth. This is corroborated by the findings of Putra et al. (2019) and Sulemana et al. (2021), who recorded higher maize growth following compost application. The fact that composts 3, 2, and 4 had similar effects on the plant height of some cashew varieties as composts 5 and 6 suggests that a combination of cashew apple residue, biochar, and poultry manure can serve as an equally effective source of plant nutrients. The rationale behind this similarity lies in the consistent impact these amendments had on the compost. Additionally, this approach has the potential to contribute to soil carbon sequestration and provides a method for managing cashew apple waste while supplying organic fertilizer. This was corroborated by similar plant growth in the second year, regardless of the compost type used.

In summary, when considering nut yield, the high basic cation contents in compost types 3 and 6, along with the high‐yielding characteristics of B3T57, likely account for the greater nut outputs observed with these compost treatments. Rupa et al. (2013) reported similar increases in nut yield following potassium fertilizer application in cashew cultivation. Thus, even with less vegetative growth in terms of stem girth and height compared to B3T101 or B3T80, B3T57's superior nut yield suggests a strategic allocation of resources favouring reproductive development over biomass accumulation under these compost conditions.

## 5 Conclusion

Cashew apple residue can be used as composting material, especially when combined with biochar, providing an alternative strategy for enriching the fragile soils of Northern Ghana. Different cashew varieties responded differently to the compost applications, highlighting the unique varietal nutrient requirements and the need to tailor fertilizer types or amendment regimes that will best meet the nutrient requirements of the different varieties released to farmers.

## Funding

This work was supported by Cocoa Research Institute of Ghana.

## Conflicts of Interest

The authors declare no conflicts of interest.

## Supporting information


**Table S1:** Effects of compost application on the growth parameters of cashew varieties over two growing seasons.

## Data Availability

The data that support the findings of this study are available in the Supporting Information.
