# Supplementary material for: Biochar‐Compost From Cashew Apple Residue as a Soil Amendment for Cashew Cultivation in Ghana
Source: Plant Environ Interact. 2026 Mar 10;7(2):e70138. doi: 10.1002/pei3.70138 (PMC12973148; doi:10.1002/pei3.70138)
Supplement: Supplementary file 1 — Table S1: Effects of compost application on the growth parameters of cashew varieties over two growing seasons. [file PEI3-7-e70138-s001.docx]

Supplementary Table 1: Effect of compost application on growth parameters of the cashew varieties over two growing seasons

| Reps | Variety | Compost type | Diameter | Height | Diameter | Height |
| --- | --- | --- | --- | --- | --- | --- |
|  |  |  | cm | | | |
|  |  |  | 2023 | | 2024 | |
| 1 | B3T57 | 1 | 0.37 | 118.47 | 0.65 | 194.1 |
| 2 | B3T57 | 1 | 0.46 | 164.4 | 0.71 | 198.7 |
| 3 | B3T57 | 1 | 0.33 | 134.1 | 0.61 | 173.1 |
| 1 | B3T57 | 2 | 0.46 | 182.4 | 0.73 | 214.1 |
| 2 | B3T57 | 2 | 0.43 | 167.5 | 0.67 | 198.2 |
| 3 | B3T57 | 2 | 0.35 | 145.53 | 0.69 | 208.9 |
| 1 | B3T57 | 3 | 0.4 | 148.03 | 0.67 | 190.3 |
| 2 | B3T57 | 3 | 0.38 | 107.7 | 0.59 | 152.7 |
| 3 | B3T57 | 3 | 0.34 | 141.1 | 0.73 | 232.8 |
| 1 | B3T57 | 4 | 0.42 | 114.7 | 0.49 | 158.7 |
| 2 | B3T57 | 4 | 0.41 | 169.03 | 0.71 | 213.4 |
| 3 | B3T57 | 4 | 0.38 | 144.17 | 0.7 | 216.8 |
| 1 | B3T57 | 5 | 0.38 | 157.27 | 0.66 | 211.3 |
| 2 | B3T57 | 5 | 0.38 | 157.83 | 0.73 | 230.2 |
| 3 | B3T57 | 5 | 0.4 | 152.87 | 0.68 | 184.9 |
| 1 | B3T57 | 6 | 0.57 | 169.27 | 0.69 | 212.9 |
| 2 | B3T57 | 6 | 0.5 | 159.8 | 0.78 | 218.4 |
| 3 | B3T57 | 6 | 0.35 | 148.17 | 0.75 | 236.8 |
| 1 | B3T101 | 1 | 0.54 | 151.57 | 0.85 | 202.2 |
| 2 | B3T101 | 1 | 0.5 | 155.6 | 0.78 | 218.6 |
| 3 | B3T101 | 1 | 0.45 | 150.87 | 0.82 | 224.5 |
| 1 | B3T101 | 2 | 0.54 | 191.27 | 0.92 | 269 |
| 2 | B3T101 | 2 | 0.43 | 159.57 | 0.76 | 220.2 |
| 3 | B3T101 | 2 | 0.48 | 155.3 | 0.74 | 220.6 |
| 1 | B3T101 | 3 | 0.53 | 187.23 | 0.77 | 236.9 |
| 2 | B3T101 | 3 | 0.6 | 210.73 | 0.81 | 262.1 |
| 3 | B3T101 | 3 | 0.48 | 181.03 | 0.51 | 158.2 |
| 1 | B3T101 | 4 | 0.57 | 165.67 | 0.81 | 211 |
| 2 | B3T101 | 4 | 0.54 | 162.33 | 0.8 | 246 |
| 3 | B3T101 | 4 | 0.42 | 128.2 | 0.64 | 180.2 |
| 1 | B3T101 | 5 | 0.51 | 208.63 | 0.76 | 221.6 |
| 2 | B3T101 | 5 | 0.52 | 192.83 | 0.82 | 244.2 |
| 1 | B3T101 | 6 | 0.48 | 154.83 | 0.83 | 227.2 |
| 2 | B3T101 | 6 | 0.46 | 174.23 | 0.74 | 217.2 |
| 3 | B3T101 | 6 | 0.55 | 170.93 | 0.87 | 243.9 |
| 1 | B3T80 | 1 | 0.48 | 161.77 | 0.68 | 204.4 |
| 2 | B3T80 | 1 | 0.47 | 160.63 | 0.78 | 223 |
| 3 | B3T80 | 1 | 0.51 | 154.5 | 0.73 | 213.4 |
| 1 | B3T80 | 2 | 0.43 | 171.13 | 0.76 | 219.1 |
| 2 | B3T80 | 2 | 0.47 | 202.87 | 0.78 | 238.9 |
| 3 | B3T80 | 2 | 0.49 | 203.53 | 0.76 | 225.5 |
| 1 | B3T80 | 3 | 0.46 | 143.83 | 0.75 | 210.8 |
| 2 | B3T80 | 3 | 0.52 | 205.8 | 0.93 | 237.9 |
| 3 | B3T80 | 3 | 0.49 | 187.47 | 0.85 | 250.8 |
| 1 | B3T80 | 4 | 0.45 | 163.6 | 0.69 | 208.3 |
| 2 | B3T80 | 4 | 0.47 | 196.8 | 0.78 | 233.5 |
| 3 | B3T80 | 4 | 0.37 | 168 | 0.75 | 225 |
| 1 | B3T80 | 5 | 0.5 | 171.43 | 0.77 | 214.4 |
| 2 | B3T80 | 5 | 0.39 | 161.2 | 0.64 | 212.2 |
| 3 | B3T80 | 5 | 0.47 | 171.53 | 0.73 | 218.9 |
| 1 | B3T80 | 6 | 0.42 | 170.23 | 0.69 | 210.6 |
| 2 | B3T80 | 6 | 0.44 | 175.83 | 0.71 | 245.2 |
| 3 | B3T80 | 6 | 0.48 | 189.17 | 0.81 | 234.8 |
